# Earwig-inspired foldable origami wing for micro air vehicle gliding

**DOI:** 10.3389/frobt.2023.1255666

**Published:** 2023-11-09

**Authors:** Risa Ishiguro, Takumi Kawasetsu, Yutaro Motoori, Jamie Paik, Koh Hosoda

**Affiliations:** ^1^ Adaptive Robotics Laboratory, Division of Systems Science, Department of Systems Innovation, Graduate School of Engineering Science, Osaka University, Toyonaka, Japan; ^2^ Fluid Mechanics Group, Graduate School of Engineering Science, Osaka University, Toyonaka, Japan; ^3^ Reconfigurable Robotics Laboratory, Institute of Mechanical Engineering, School of Engineering, Ecole Polytechnique Federale de Lausanne, Lausanne, Switzerland

**Keywords:** MAV, foldable wing, wind tunnel experiment, origami, insect-inspired

## Abstract

Foldable wings serve as an effective solution for reducing the size of micro air vehicles (MAVs) during non-flight phases, without compromising the gliding capacity provided by the wing area. Among insects, earwigs exhibit the highest folding ratio in their wings. Inspired by the intricate folding mechanism in earwig hindwings, we aimed to develop artificial wings with similar high-folding ratios. By leveraging an origami hinge, which is a compliant mechanism, we successfully designed and prototyped wings capable of opening and folding in the wind, which helps reduce the surface area by a factor of seven. The experimental evaluation involved measuring the lift force generated by the wings under Reynolds numbers less than 2.2 × 10^4^. When in the open position, our foldable wings demonstrated increased lift force proportional to higher wind speeds. Properties such as wind responsiveness, efficient folding ratios, and practical feasibility highlight the potential of these wings for diverse applications in MAVs.

## 1 Introduction

Micro air vehicles (MAVs) offer versatile solutions for transportation, measurement, and communication tasks (Floreano and Wood, 2015). Foldable wings (Dileo and Deng, 2009; Ma et al., 2013; Liu et al., 2016; Zou et al., 2016; Faber et al., 2018; Chen et al., 2019) have been increasingly used in MAVs with fixed- or flapping-wing configurations because they enable size reduction during transportation while preserving effective flight capabilities (Dufour et al., 2016). This further facilitates the simultaneous transportation of multiple MAVs, enabling large-scale applications such as extensive search operations (Floreano et al., 2017). Researchers have drawn inspiration from birds, bats, and insects to develop foldable wing designs, which have progressed remarkably in recent years (Muhammad et al., 2010; Truong et al., 2014; Dufour et al., 2016; Baek et al., 2020; Li et al., 2021; Saito et al., 2021). The folding ratio, which quantifies the ratio between unfolded and folded wing areas, is an important factor in the evaluation of the performance of foldable wings (Muhammad et al., 2010). Higher folding ratios enable greater wing extension, resulting in larger wing areas when unfolded and increased lift forces. Several representative examples include the beetle-inspired wings with a folding ratio of six (Truong et al., 2014) and a ladybird-inspired glider module with a folding ratio of eight (Baek et al., 2020). These advancements in foldable wing design, coupled with biomimetic inspiration, contribute to the development of the MAV technology.

In the development of foldable wings, a trade-off exists between increasing the folding ratio and decreasing the weight of the foldable wing. Previously, pin joints were typically used in small foldable wings (Muhammad et al., 2010; Truong et al., 2014; Li et al., 2021). According to Wagner et al. (Wagner et al., 2020), a pin joint provides a single rotational degree of freedom with a cylindrical stud that connects two links. Using pin joints is an effective way to maintain wing stiffness and keep the wing open in the wind. However, using pin joints to achieve complex folding with a high folding ratio, such as that of an earwig, can result in a heavier and thicker wing. This is because several links and pins are required to achieve multiple folds with pin joints.

This study attempted to resolve the aforementioned trade-off using the folding structure of earwig wings and the origami hinge. We imitated the folding structure of earwig wings to improve the folding ratio of the folded wings. As mentioned previously, earwigs have the highest folding ratio among insects (Deiters et al., 2016), and their folding design has recently been elucidated (Saito et al., 2020). We used an origami hinge design (Zuliani et al., 2018) to achieve a high folding ratio while developing thin and lightweight wings. The flexibility of sheet-like materials was emulated in the origami design to achieve folding. Therefore, the proposed solution helps obtain thin and lightweight wings with multiple folds. Additionally, the developed solution yields a relatively smaller wing that is simple to assemble (Wagner et al., 2020). The origami design is suitable for reproducing the folding structure of earwigs consisting various overlaps when folded.

Earwig wings have a distinctive fan-shaped accordion-like folding mechanism, and this unique folding is crucial because it enables folding with a high folding ratio (Saito et al., 2020). Unlike birds and bats, which use joint hinges and end up with thicker folded wings (Muhammad et al., 2010; Marks et al., 2013; Jitsukawa et al., 2017), earwigs can keep their wings thin thanks to this folding style. In fan-shaped accordion folding, origami hinges are used. The fold of a joint hinge, that is, the pin part, is perpendicular to the wing surface, whereas that of an origami hinge is parallel to the wing surface. Origami hinges are prone to buckling under the force received from the air. Therefore, it is necessary to confirm that the foldable wing with origami hinges is actually practical in the wind. Additionally, for practical purposes such as machines or robots, actuators are required to control the folding and unfolding of the wings, even though certain previous studies tried to copy how earwig wings fold without actuators (Mintchev et al., 2018).

We have successfully developed artificial wings with folding ratios of approximately five and seven, and we have imitated the characteristic folding structure of earwigs with an origami hinge design. We evaluated the performance of the fabricated folding wings based on wind tunnel experiments from two perspectives: first, the ability of the wings to open and close in the wind, and second, the aerodynamic performance of the wings in the unfolded state. To evaluate the aerodynamic performance of the wings, we measured the lift force produced by the wing in the open state and calculated the magnitude of the lift relative to the drag force. The wind tunnel experiments confirmed that the developed wings could open and fold at a wind speed of 4 m/s. We also confirmed that the wing produced a higher lift force when opened than when folded. Furthermore, lift-drag (LD) ratio of the wing with a folding ratio of approximately five was greater than one for all tested wind speeds.

## 2 Earwig-inspired foldable origami wing

We developed a wing that partially reproduces the hindwing folding in earwigs to obtain a foldable wing with a high folding ratio. We employed an origami hinge to reproduce the earwig folding. Additionally, we used pouch motor actuators to develop thin and lightweight wings. A detailed description of the fabricated wings is given below. First, we provide a detailed explanation of the earwig wing folding mechanism. Second, we explain the thin origami hinge design of the proposed wing solution. Third, the lightweight pouch motor actuator used to unfold the wings and the motor actuator used to fold the wings are discussed. Further, we explain the materials used for the wings and the manufacturing method. Finally, the wing specifications are described.

### 2.1 Folding design of earwig hindwing

**FIGURE 1:**
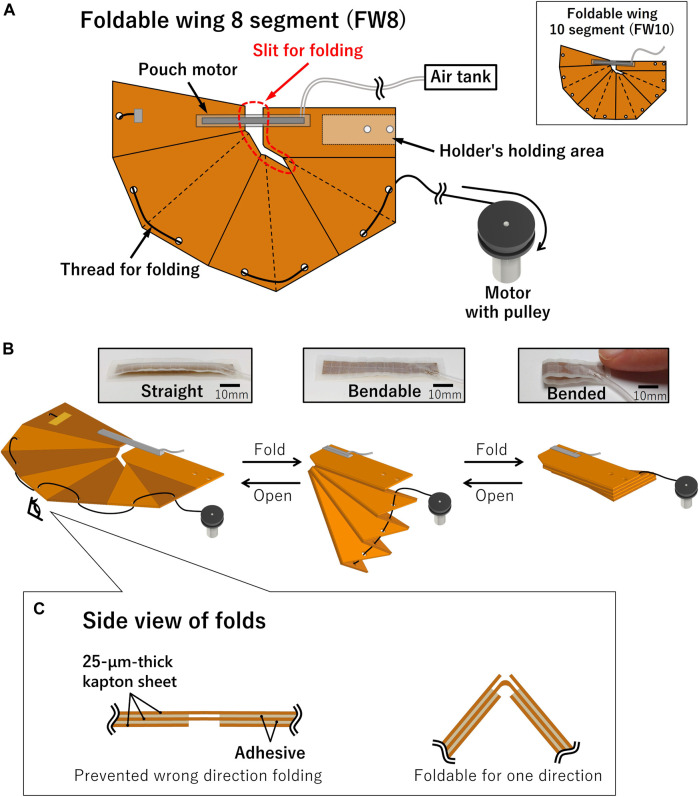
Schematic view of fabricated artificial wing. **(A)** Overall view of one wing of artificial wing. Pouch motor is incorporated as actuator to open wing, and thread and motor are incorporated as actuators to close wing. Continuous and dashed lines indicate mountain and valley folds, respectively. In FW10, the wing segments become 10. **(B)** Schematic diagram of opening and closing of fabricated artificial wing and photos of pouch motor. Air is injected into the pouch motor while the thread is loose, and pouch motor straightens to open wing; when the air is released from the pouch motor and wing can bend, the motor pulls thread to close wing. **(C)** Design of folds. Direction of fold may be determined by changing design of surface layer material.

We fabricated a foldable artificial wing (Figure 1) based on the folding structure of an earwig hindwing (Saito et al., 2020). It should be noted that earwigs have high-folding-rate wings, with folding rates as high as 10 (Haas et al., 2000) and up to 18 in certain species (Deiters et al., 2016). Following the method described in (Saito et al., 2020), a folded structure of the earwig hindwing can be obtained by varying the number of folds and the angle of folds. The folding of the earwig’s wing can be divided into two main steps: fan-shaped accordion folding and folding in half. Folding with a ratio of approximately five or nine can be achieved in the first folding. The second folding can yield a folding ratio of approximately two, resulting in a folding ratio of 10 in total. In other words, fan-shaped accordion folding is expected to achieve a high folding ratio of 5–9. In this study, we reproduced the first folding structure, fan-shaped accordion folding.

The region of the wing enclosed by the fold lines or outline edge is referred to as “a segment”. To analyze the change in performance in a fluid according to the change in the number of folds, we designed a wing with eight segments (Foldable wing 8; FW8) and a wing with ten segments (Foldable wing 10; FW10).

### 2.2 Origami hinge design

We designed a foldable wing using an origami hinge. Origami hinges are made of layered sheets of material. Here, bending is achieved using the material’s flexibility. The hinge part is often thicker than the non-hinge part in pin-joint hinges. However, with origami hinges, the thickness can be reduced to a value similar to that of the material, resulting in a hinge thickness equal to or less than that of the non-hinged part.

In addition, the wing developed in this study was designed to fold in only one direction (Figure 1C). In this design, the one-side surface layers (bottom side in Figure 1C) were partially removed at the folds, and thus, they did not interfere with each other when folded. In contrast, the other surface layers (Top side in Figure 1C) collided at the folds. Therefore, folding did not occur in the direction of the collision of the surface layers; rather, it occurred solely in the direction where the surface layers did not coincide. This design assists in controlling the entire folding motion with only one folding actuator.

### 2.3 Thin and lightweight pouch motors as wing-opening actuator

**FIGURE 2:**
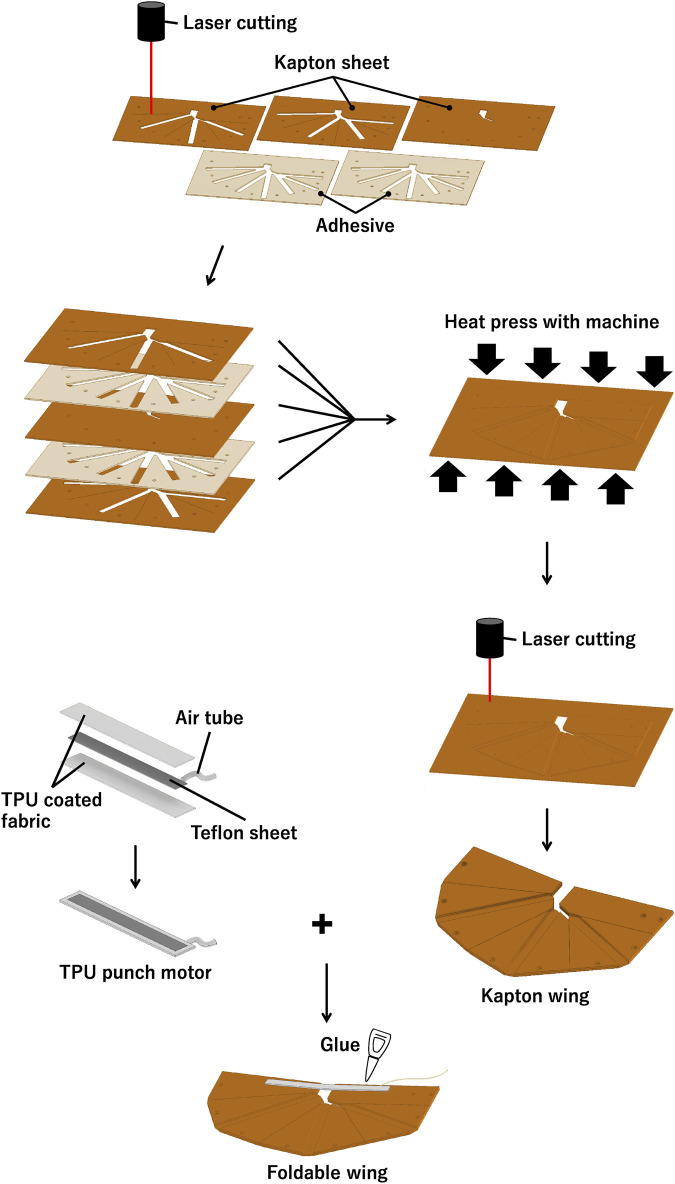
Wing fabrication. Kapton sheets and adhesives are cut out according to design, arranged in layers, and heat-pressed to adhere them together. After that, laser cutter cuts out the external shape, and pouch motor is attached with adhesive.

We used a pneumatic actuator, referred to as a pouch motor, (Niiyama et al., 2015; Robertson et al., 2021), to open the wings (Figure 1B). A pouch motor inflates and increases the rigidity when filled with air. When the air is released, the pouch motor retains the sheet-like shape and folds up. When not inflated, the pouch motor is thin, resulting in thinner wings when folded. Moreover, the pouch motor allows the opening actuation part to be lightweight. Thus, the weight of this type of actuator does not prevent the wings from opening. We fabricated the pouch motor using a TPU-coated fabric (N6.6 20D lattice/TPU, Jiaxing-shi Yingcheng Fangzhi Co., Ltd.). The thickness of the TPU-coated fabric was 0.06 mm. As shown in Figure 2, a Teflon sheet with a small surface area was placed between two TPU-coated fabrics, and pressure was applied while heating at 190°C to ensure that the TPU-coated fabrics adhered to each other. Because Teflon and TPU generally do not adhere to each other, the assembly functions as a pouch motor by allowing air to enter the Teflon portion. In this study, a pouch motor with a 7-mm-wide and 50-mm-long Teflon part was fabricated and used for wing opening. The thickness of the fabricated pouch motor, when it was not inflated, was 0.250 mm. When not inflated, the pouch motor essentially comprises two layers of TPU-coated fabrics and one Teflon sheet, which is highly pliable and does not inhibit the actuation of closing the wings, as described below. The Supplementary Video S1 shows the unfolding and folding operations.

We fabricated a slit in the wing-body to unfold it from the fully folded state (Figure 1A). This slit also prevented interference between the wings and the pouch motor when the wings were completely folded. A small motor (SM8-F20, Minebea Co., Ltd.) was used to fold the wings. A hole was formed in each wing segment, and the motor reeled a thread through the hole to achieve folding (Figure 1B). When opening the artificial wing, the voltage input to the motor was set to zero. Air was continuously input to the pouch motor at 1.5 bar. The voltage input to the motor and the air pressure input to the pouch motor were changed manually. Further, the switch for the voltage input to the motor was operated from a PC using a microcontroller (Teensy LC, SparkFun Electronics).

### 2.4 Sheet material for artificial wings

We used 25-μm-thick kapton polyimide films (2271K41, McMaster-Carr Supply Company) and 50 μm-thick polyamide hotmelt film adhesives (POLI-MELT 701, POLI-TAPE Klebefolien GmbH) for the artificial wings. Kapton sheet material was selected for this study because it is thin, easy to fabricate, and has a high tension-allowable stress. The Young’s modulus of a single Kapton polyimide film is 3.4 GPa.

As shown in Figure 2, the artificial wing was fabricated by alternately layering three Kapton sheets and two adhesive sheets. The sheets were pressed with a heated press machine (3853CE, Carver, Inc.). It was observed that the heat press reduces the thickness of the adhesive sheet to less than 50 μm. Following adhering, we cut the outer shape with a laser cutter (Trotec Speedy 400, Trotec Laser GmbH). In particular, the actuators’ pouch motors were attached to the wings.

### 2.5 Overview of the assembled wings

**FIGURE 3:**
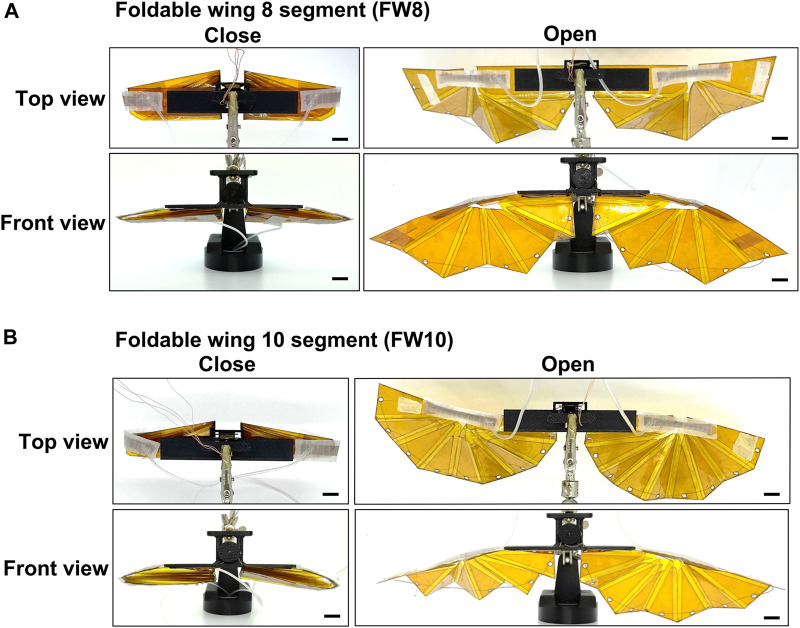
Wing photos of artificial foldable wings. **(A)** The eight-segment foldable artificial wing (FW8). **(B)** The ten-segment foldable artificial wing (FW10). All black scale bars indicate 10 mm.

Figure 3 shows a photograph of the fabricated artificial wing. The left and right wings were clipped using gripping parts fabricated using an FDM 3D printer (uPrint SE, Stratasys Ltd.).

Table 1 lists the fabricated airfoils’ mass, thickness, and wing area values. We measured the mass using a digital weight scale (400-TST008, Sanwa Supply Inc.). The thickness was measured using a digimatic outside micrometer (MDC-25MX 293-230-30, Mitutoyo Corporation.). The wing area was calculated according to the data for wing fabrication. We designed the wingspan of both artificial wings to be 125 mm for one wing when opened. The wing chord lengths of FW8 and FW10 were 93.3 and 86.4 mm (rounded to three significant digits), respectively, when opened.

**TABLE 1:** Artificial wing specifications. Area and folding ratio are rounded to three significant digits.

Wing type	Foldable wing 8 segment (FW8)	Foldable wing 10 segment (FW10)
Weight [g]	11.4	11.4
Thickness (Open state) [mm]	0.161	0.168
Thickness (Closed state) [mm]	1.327	1.673
Inflated pouch motor part thickness [mm]	4.883	4.928
Opening area size [mm2]	8,450	7,590
Folding area size [mm2]	1,690	1,110
Folding ratio	1/4.99	1/6.87

## 3 Experimental evaluation

It was necessary to verify if the foldable wing described previously could open in the wind and maintain its shape in the open state, similar to the opening of the jump glider wings (Vidyasagar et al., 2015) after jumping. This is because these wings did not possess a skeleton and comprised a sheet-like material. Lift force and LD ratios were used to evaluate wing performance. The LD ratio is calculated as the lift force divided by the drag force. The drag force is a force acting on the wing in the opposite direction parallel to the movement direction. We observed that the wing functions properly when unfolded if it generates a difference in the lift force before and after the unfolding. In addition, a higher LD ratio implies a higher gliding performance. This is because the larger the LD ratio, the farther the wing reaches in a glide. When the LD ratio is greater than one, the wing can glide at a glide angle of 45° or more. We conducted two experiments on the fabricated artificial wings in this study. The first experiment involved opening of the wings in the wind. The second one was conducted in a wind tunnel to measure the lift and drag forces and to verify the LD ratio.

In the first opening experiment, we used FW8. The variables in the experiment included varying wind speeds of 0, 1, 2, and 4 m/s. The results confirmed that the wings could be opened even at low and high wind speeds. This result was confirmed by checking whether the opening and folding were possible at each wind speed. Moreover, we measured the lift force before, during, and after opening to confirm that the lift force changed due to the opening. During the experiment, the wind flowed from the leading edge to the trailing edge of the wing, given an angle of attack of 0°. The wings were held in the folded and open states for a minimum of 10 s each. We generated wind at each velocity, calibrated the sensors with the wings folded, and then conducted the measurements.

In the second wind tunnel experiment, we investigated the lift and drag forces generated by the wind on the fabricated artificial wing during the opened state. The number of wing segments and wind speed were the variables in this experiment. In particular, wings with 8 and 10 segments, namely, FW8 and FW10, respectively, were used. In addition, similar to the first experiment, the wind speeds of 0, 1, 2, and 4 m/s were used. During the experiment, the wind flowed from the leading edge to the trailing edge of the wing. The angle of attack was 0°. When measuring the forces, the artificial wing was motionless in the closed and opened states for 5 s each, and an average of the 5 s measurements was used as the force value. Before the measurements, the sensors were calibrated for each FW8 and FW10 at a wind speed of 0 m/s and with the wing closed. We also conducted the same experiment with the wings closed. The Reynolds number at each wind speed is listed in Table 2. For a detailed comparison, we conducted the same experiment using a fixed wing with the same wing area as the FW8 in the closed state. We fabricated this wing using 0.2-mm-thick fiber-glass sheets (200 μm FR4, Masterpatex e.K.) and used the same wing holder as the foldable wing.

**TABLE 2:** Reynolds number at each wind speed, rounded to two significant digits.

Wind speed [m/s]	Reynolds number
0	0.0
1	5.6 × 10^3^
2	1.1 × 10^4^
4	2.2 × 10^4^

**FIGURE 4:**
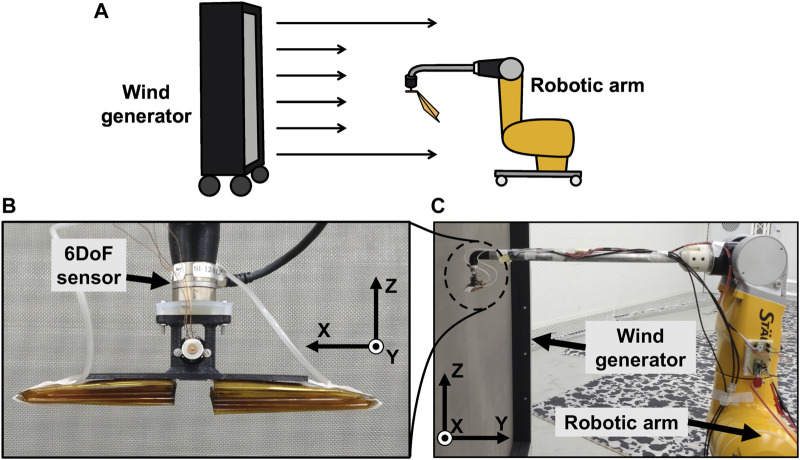
Wind tunnel experiment setup. **(A)** Schematic diagram of entire experimental environment. **(B)** Photograph of area around sensor. Observed from body side of the robot arm toward wind tunnel. **(C)** Photograph of robot arm. Photo was taken from side of robot arm.

Figure 4 illustrates the experimental setup for all the wind tunnel experiments. The wind module (WindShape Ltd.) was used to generate a laminar flow with a cross-section that was 2-m-wide and 1.75-m-high. In the laminar flow, an artificial wing attached to the tip of the force sensor (Nano17 SI-12-0.12, ATI Industrial Automation) was placed using a robotic arm (TX2-90, Staubli International AG) (Figure 4B). The sensor was attached to the end of a metal pipe attached to the robot arm via a part fabricated using the FDM 3D printer (uPrint SE, Stratasys Ltd.). The sensor was placed at a distance of 20 cm from the wind module. The sampling rate of the sensor was 20 Hz. Moreover, the air pressure to actuate the pouch motor for wing opening was 0.2 MPa in all experiments. This deflection angle of the foldable wing was measured using Kinovea (Charmant and Contributors, 2021) from the video recorded during the experiment.

## 4 Result

This section explains the outcomes of our experiments conducted in the wind tunnel. Initially, we present the experimental results that confirmed the ability of our foldable wings to open and fold in the wind. Then, we present the outcomes of another experiment that aimed to measure the lift and drag generated by the open foldable wings in the wind.

**FIGURE 5:**
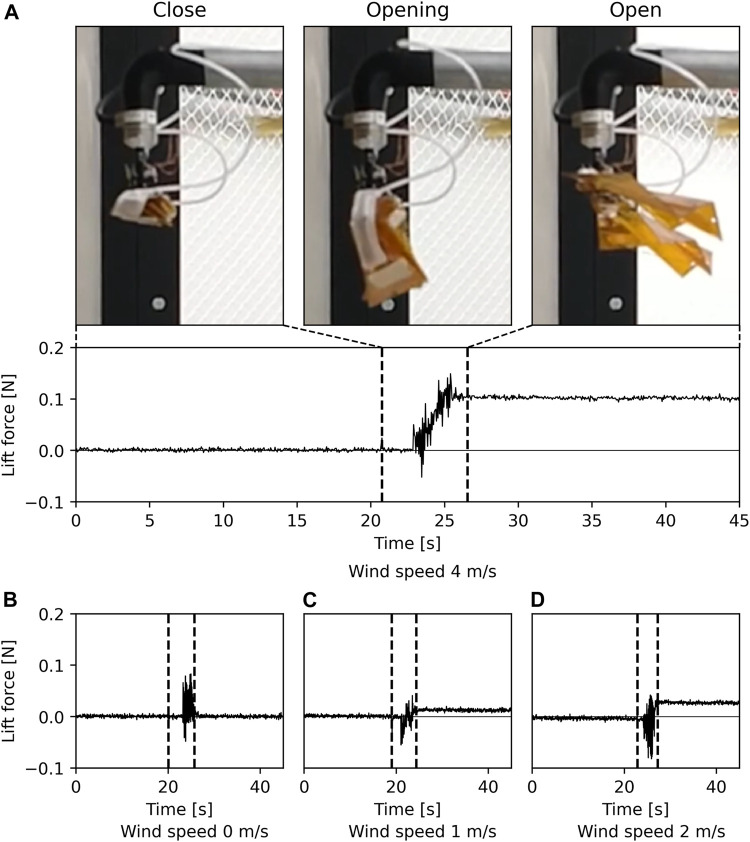
Results of experimental opening of an artificial wing in wind. Graphs show lift force at each wind speed, with openings between the dashed lines. Photographs show the wing in a folded state, in the middle of an opening, and opened state at a wind speed of 4 m/s, respectively. **(A)** Sensor results of lift force at a wind speed of 4 m/s, and photographs of the wing in each state. Sensor results of lift force at a wind speed of **(B)** 0 m/s, **(C)** 1 m/s, and **(D)** 2 m/s.

Both FW8 and FW10 successfully opened and folded in the wind at all wind speeds (0, 1, 2, and 4 m/s). The folding ratio was 4.99 for FW8 and 6.87 for FW10. Figure 5 A, B, C, and D illustrate the lift changes during opening and folding at wind speeds of 4, 0, 1, and 2 m/s, respectively. The horizontal and vertical axes represent time and the lift force measured by the sensor. A change in the lift force after opening can imply that the artificial wing achieved a considerable change in the wing area for gliding. The opening was performed in the area between the vertical dotted lines in the graph. The top three photographs illustrate the wing in the closed, opening, and opened states at a wind speed of 4 m/s. The measured vertical force is positive in the upward direction, and the lift force is generated when the value of the vertical axis is greater than zero. In particular, a change in the value of the lift force after opening was observed at all wind speeds except 0 m/s. The higher the wind speed, the greater lift force produced after opening.

**FIGURE 6:**
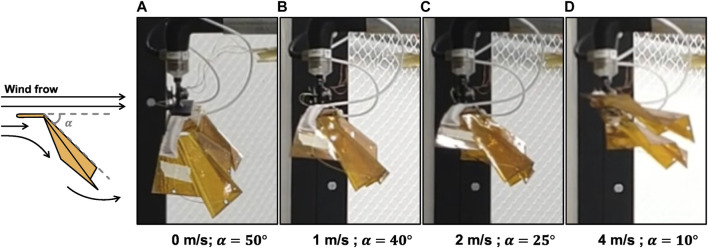
FW8 artificial wing in opened state at each wind speed. At the same time, deflection angle α of the wing’s trailing edge is shown. Situation at a wind speed of **(A)** 0 m/s, **(B)** 1 m/s, **(C)** 2 m/s, and **(D)** 4 m/s.

Figure 6 shows the state of the foldable wing FW8 at each wind speed. The angle α of the trailing edge of the wing decreases as the wind speed increases. As explained in section 3, the angle α obtained in this experiment was regarded as the effective attack angle.

**FIGURE 7:**
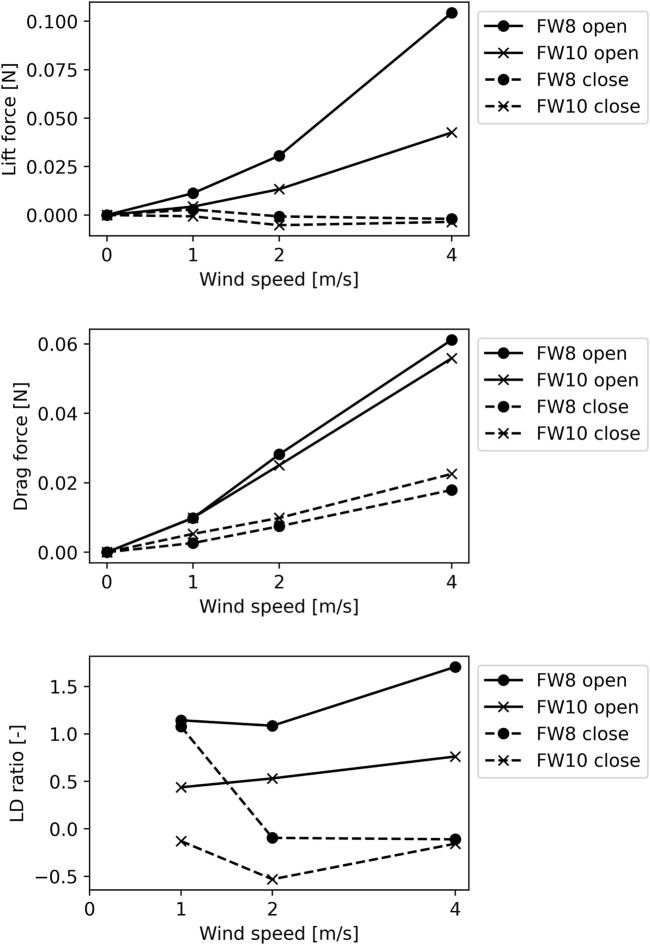
Lift, drag, and lift-drag ratio (LD ratio) results from wind tunnel tests. We investigated lift and drag forces generated by foldable wing with eight folds or edges (FW8) and foldable wing with ten folds (FW10) in open and closed states, respectively. LD ratios were calculated from results. In all graphs, horizontal axis indicates wind speed.

We conducted wind tunnel experiments to investigate the lift and drag forces generated by each foldable wing in the opening and closed state at each wind speed. The graph in Figure 7 shows the lift force, drag force, and the LD ratio of the artificial wing in the wind tunnel experiment. The horizontal axis represents the wind speed, while the vertical axis represents the lift force, drag force, and LD ratio values. The LD ratio is omitted in the case of zero wind speed because the drag force is zero. Table 3 lists each foldable wing’s lift and drag coefficients at each wind speed, which are calculated from the experimental results. We used the wing area in the unfolded state in the calculation. Each coefficient varies with each wind speed because the wings are flexible and deform with wind speed.

**TABLE 3:** Lift and drag coefficients for each foldable wing at each wind speed, calculated from experimental results, and rounded to three significant digits. Wing area in unfolded state was used in calculation.

	FW8		FW10	
Wind speed [m/s]	CL	CD	CL	CD
1	2.06	1.80	0.884	2.02
2	1.40	1.29	0.679	1.28
4	1.19	0.700	0.542	0.711

**FIGURE 8:**
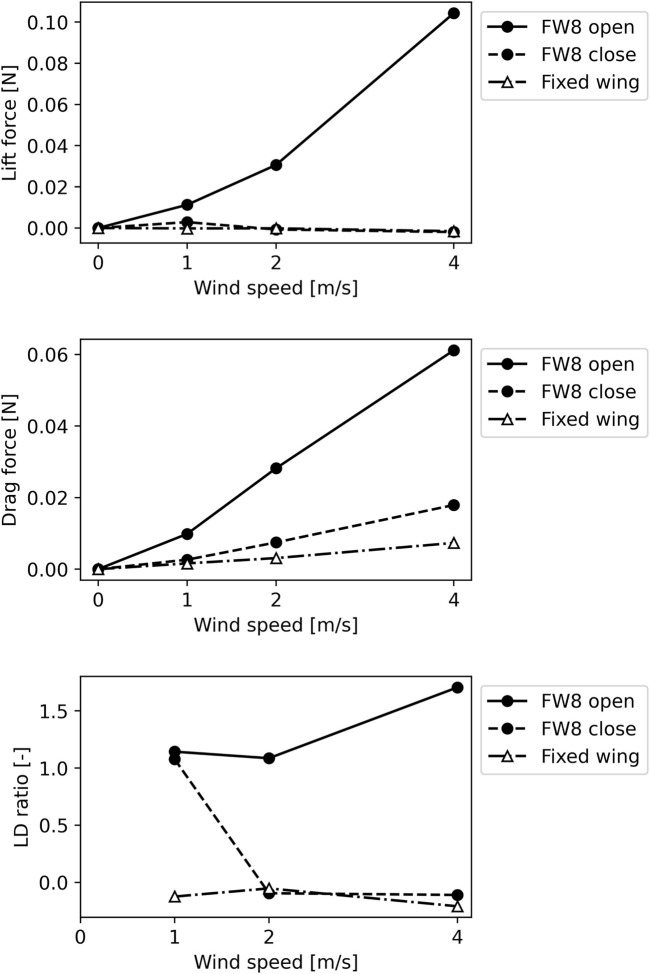
Lift, drag, and lift-drag ratio (LD ratio) results of foldable wing and fixed wing. We investigated lift and drag forces generated by foldable wing with eight folds or edges (FW8) in open and closed states. LD ratios were calculated from results. Same experiments were also conducted on fixed wing with same wing area as FW8 in closed state for comparison. In all graphs, horizontal axis indicates wind speed.

The open wing exhibited a higher lift and LD ratio than the closed wing. The wind tunnel experiment results showed that the lift drag forces increased with the wind speed. In particular, FW8 generated a higher lift force than FW10. The LD ratio of FW8 exceeded 1.00 for all wind speeds. We compared FW8, which produced a greater lift force in the unfolded state than FW10, with a fixed wing with the same wing area as FW8 in the closed state (Figure 8). The FW8 in the open state exhibited a higher lift force and LD ratio than the fixed wing.

## 5 Discussion

This study presented a method for fabricating foldable wings based on the folding structure of earwig hindwings. We developed a foldable wing that imitated a part of the folding structure of the earwig’s hindwing and folded to approximately one-fifth of the original area using origami hinges. The earwig’s wing folds into a fan shape around the center of the wing’s leading edge and then folds further into about half. For creating a foldable wing that imitates the earwig’s wing folding structure, the feasibility of fabricating a wing with this fan-shaped folding structure is important, and it is necessary to confirm the essential aerodynamic performance. Therefore, this study established a method to facilitate the fan-shaped folding of the unique folding structure of earwig’s wings. We successfully achieved the first folding stage. However, achieving the second stage of folding was challenging. Thus, overcoming this challenge to simultaneously implement the first and second stages of earwig wing folding will help develop an artificial wing that can fold with a folding ratio of approximately 10, equivalent to that of an actual earwig wing.

The FW8 wing fabricated in this study could open its wings in the wind and exhibited greater lift force and LD ratios in the deployed state than fixed wing with the wing area of folded FW8. This foldable wing can produce more value than a fixed wing when unfolded. Both foldable wings exhibited greater lift force and LD ratios in the unfolded state than in the folded state. This indicates that unfolding improves the wing’s functionality as a wing. The LD ratio of the FW8 wing exceeded one at all wind speeds. These results suggest that the FW8 wing could glide. In addition, the flexibility in the wing span direction may explain why FW10 generated a smaller lift force than FW8 in this study. The FW10 has more origami hinges than FW8, which increases the wing’s flexibility. Because the force of the pouch motor to open the wing’s leading edge is the same for FW8 and FW10, the center of the wing’s trailing edge deflects more easily for FW10, which has more hinges. When the wing deflects, the lift force may decrease because the size of the wing’s projected area, viewed from a direction perpendicular to the wind flow, becomes smaller. The increased folding ratio could be a trade-off for wing performance.

This study demonstrates the feasibility of a foldable wing based on an earwig’s folding structure and it is potential for use as a gliding wing. This folding method can easily change parameters such as the number of folds by imitating the folding structure of earwig wings mainly due to the recent progress made in understanding this structure. Folded wings, which allow easy modification of detailed design parameters, can be easily optimized to suit the situation of the opened object. The wing shape used in this study is semi-circular, similar to the earwig hindwing. The folding method used in this study does not limit the wing outline, which helps the user choose any wing outline. It is known that the wing outline directly affects wing performance. Because this study aims to design and realize the folding function of a folding wing, we hypothesize that there is potential in optimizing airfoil shape and wing performance in future research. Further, the folding structure of earwig wings could be applied to artificial wings and the opening of membrane components, such as solar panels on satellites. However, as the root of the wing is the only part holding the wing in place during opening, a detailed analysis regarding the deflection of the entire wing is necessary. Because the hinge portion must be fabricated from a thin and soft material to enable folding, a structure that can adequately support the hinge portion during opening is required to increase the scope of applications.

It should be noted that the fabrication performance may be an obstacle when increasing the folding ratio because incorporating more folds will reduce the size of each segment. The folding rate is limited by the performance of the processing equipment and the processing method, especially for small wings. Furthermore, Figure 7 shows that increasing the amount of folding could reduce the lift force and subsequently, the LD ratio. Excessive wing flexibility may worsen the LD ratio. Therefore, in future studies, improving the hinge part may alter the overall performance of the wing. However, increasing the stiffness of the hinge area may simultaneously inhibit folding and alter durability. This paper provides one example of balancing folding and wing performance. In addition, the hinges in this paper stayed intact even after 15 openings and closings.

In this study, we focused on gliding and did not consider wing flapping. As earwigs are flying insects that flap their wings, our wings could also have flapping potential. However, during the flapping phase, the wing can buckle at the folds, which may be detrimental to the flapping flight. There are several opinions on the ideal wing flexibility for flapping, as discussed in previous studies (Nakata et al., 2011; Kang and Shyy, 2013; Ryu et al., 2019). Therefore, we plan to conduct further research on whether our wings cater to flapping flight.

## 6 Conclusion

In this study, we investigated the aerodynamic performance of foldable wings by prototyping and studying two types of foldable wings with different folding numbers and evaluated their aerodynamic performance in a wind tunnel experiment. We developed an artificial wing that could be opened and folded only by an actuator mounted at the root of the wing with a folding ratio of approximately five or seven. The proposed artificial wing yielded a folding performance of approximately half of that of an earwig wing. Future studies may help improve the folding ratio by imitating the entire earwig wing structure.

## Data Availability

The raw data supporting the conclusions of this article will be made available by the authors, without undue reservation.
