# Supplementary material for: Earwig-inspired foldable origami wing for micro air vehicle gliding
Source: Front Robot AI. 2023 Nov 9;10:1255666. doi: 10.3389/frobt.2023.1255666 (PMC10665516; doi:10.3389/frobt.2023.1255666)
Supplement: Supplementary file 1 [file DataSheet1.PDF]

# Supplementary Material

## 1 VERIFICATION OF RIGID FLAT PLATE WING PERFORMANCE FOR COMPARISON TO FOLDABLE WING PERFORMANCE

In this study, we fabricated foldable wings using flexible materials. Since this foldable wing deforms with the wind, it is necessary to solve a fluid-structure interaction problem to model the entire wing numerically, which is complex. Additionally, there are limitations to strictly modeling the actual wing created because such a model is subject to wing stiffness variations resulting from wing materials, fold locations, and manufacturing errors. On the other hand, a rigid, thin, flat plate is the most straightforward shape of the airfoil. It is worthwhile to know the aerodynamic performance of a rigid, thin, flat plate wing to consider the lift force, drag force, and lift-drag ratio (LD ratio) produced by the foldable wings fabricated in this study. Here, we investigate the aerodynamic performance of a rigid thin plate wing for comparison by numerical simulation.

### 1.1 Method

The numerical simulations considered an incompressible fluid around a rigid flat plate. The simulation results were compared with the results of the wind tunnel experiments on the foldable wing subjected to three wind speeds (1 m/s, 2 m/s, and 4 m/s). In the simulations, the deflected trailing edge of the wing was considered a rigid wing with a semicircular plate shape of 62.5 mm radius. We set the angle of the plate to the flow to be the value of the deflection angle of the foldable airfoil observed in the experiment with foldable wings. That is, the angles shown in the table S1. This deflection angle was measured using Kinovea (Charmant and contributors, 2021) from the video recorded during the experiment. We expressed the semicircular plate using the immersed boundary method (Breugem, 2012) and fixed it at the center of the computational rectangular box.

We imposed uniform wind speeds at the inlet and the convective condition at the exit. The standard finite difference method was used to solve the Navier-Stokes equations for an incompressible fluid. The viscous and convection terms were temporally integrated using the second-order Crank-Nicolson and third-order Adams-Bashforth methods, respectively. The numerical simulation method used to solve the fluid motions is the same as that used in the study (Motoori et al., 2022).

### 1.2 Results

The top panel of Figure S1 illustrates vortices identified by the positive isosurfaces of the second invariant of the velocity gradient tensor. The horizontal axis represents the wind speed, while the vertical axis represents the lift force, drag force, and LD ratio values. The LD ratio is omitted for the case of zero wind speed because the drag force is zero. For comparison, we have simultaneously plotted the lift, drag, and LD ratios produced by a foldable wing in the unfolded state, which is the same data as the one presented in the main document.

The wind tunnel experiment and simulation results exhibited a similar trend to wind speed variation, both showing a tendency for lift and drag forces to increase as the wind speed increased. For wind speeds of 2 m/s and 4 m/s, FW8 and FW10 exhibited greater lift forces than those in the simulations. The LD ratios of FW8 were 1.00, 0.648, and 0.630 times (rounded to three significant digits) higher than the results of the simulations performed on rigid flat plates at wind speeds of 1, 2, and 4 m/s, respectively.

## 1.3 Discussion

The lift force and LD ratio of the fabricated foldable wing exceeded those of the simulation, indicating that the aerodynamic performance of the fabricated foldable wing is superior to that of a semi-circular thin plate wing of the same size. A comparison of the LD ratios indicated that the performance of the fabricated foldable wing was approximately 60% of the simulated rigid flat plate wing. Possible reasons for the difference between the wind tunnel results and the simulation results are the flexibility of the wing and the existence of other parts of the wing. In the simulations, the airfoil is rigid, but the airfoil is flexible in the experiments. Wing flexibility may improve the aerodynamic performance of the wing (Nguyen et al., 2015). Also, the gripping parts are not taken into account in the simulations. The gripping parts increase the drag force. The cross-sectional area of the gripping part relative to the flow is approximately 580 $mm^2$. Assuming a drag coefficient of 1, the drag force at a flow velocity of 4 m/s is approximately 0.0053 N. The lower the flow velocity, the smaller this drag force becomes. However, even considering this drag force, the LD ratio of the foldable wing still exceeds the simulation results. The existence of a pouch motor, which is the actuator for deployment, may also be one of the possible reasons for the difference between the wind tunnel results and the simulation results.

A comparison between the experimental results and those obtained from simulations suggests that changes in the effective angle of attack due to the deflection of the foldable wings might result in a change in the drag force when the flow velocity changes. However, we confirmed that the simulation's changing trend with wind speed is similar to that observed in the experimental results. Note that the drag force $F_D$ was calculated using the drag coefficient $C_D$, representative velocity $U$, density of the fluid $\rho$, and representative area $S$ as follows:

$$F_D = C_D(\frac{1}{2}\rho U^2 S). \tag{S1}$$

In the simulations, we reflected only the effective angle of attack at the wing's trailing edge. If the effective angle of attack remains equal, the drag coefficient and the representative area would remain nearly the same, and the lift and drag forces would be proportional to the square of the wind speed. However, the drag results of the simulation were not proportional to the constant coefficient of the square of the wind speed. This result suggests that changes in the effective angle of attack may have affected the changes in drag force. The change in the effective angle of attack due to the wing's flexibility changes the projected area viewed from a direction parallel to the wind flow, thereby reducing the increase in drag due to increased wind speed.

# FIGURE AND TABLE

**Table S1.** Deflection angle of the foldable wing in each wing speed.

| Wind speed [m/s] | Deflection angle [deg] |
|---|---|
| 0 | 50 |
| 1 | 40 |
| 2 | 25 |
| 4 | 10 |

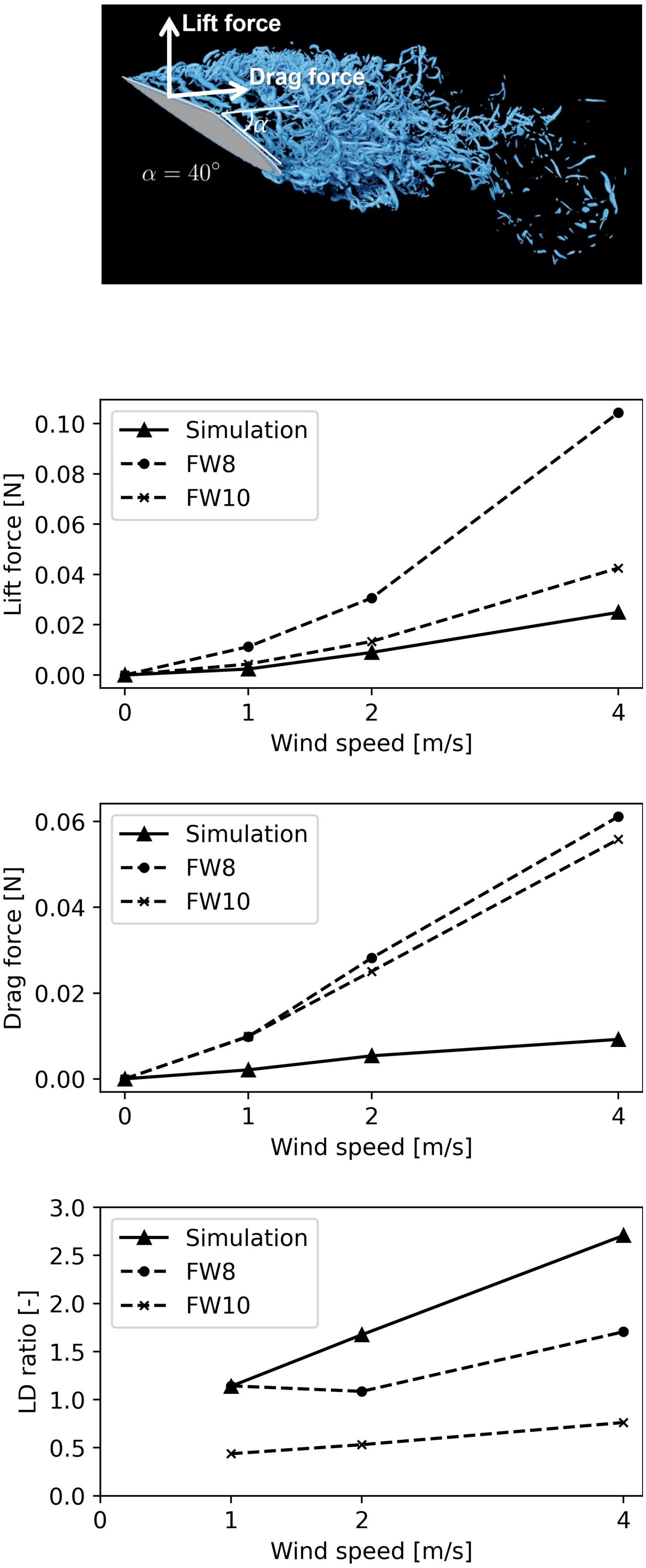


**Figure S1.** Lift, drag, and LD ratio results from wind tunnel tests and simulated on a rigid half-disc wing. In all graphs, the horizontal axis indicates wind speed. In the graphs for lift force and drag force, the vertical axis indicates the force produced by the wing in its opened state at each wind speed. In the graph for the LD ratio, the vertical axis indicates the LD ratio. The top panel shows the vortices identified by the positive isosurfaces of the second invariant of the velocity gradient tensor.

## REFERENCES

Breugem, W.-P. (2012). A second-order accurate immersed boundary method for fully resolved simulations of particle-laden flows. *Journal of Computational Physics* 231, 4469–4498

Charmant, J. and contributors (2021). Kinovea (0.9.5)

Motoori, Y., Wong, C., and Goto, S. (2022). Role of the hierarchy of coherent structures in the transport of heavy small particles in turbulent channel flow. *Journal of Fluid Mechanics* 942

Nguyen, N., Kaul, U., Lebofsky, S., Ting, E., Chaparro, D., and Urnes, J. (2015). Development of variable camber continuous trailing edge flap for performance adaptive aeroelastic wing. In *SAE AeroTech Congress & Exhibition*. ARC-E-DAA-TN25273
